# Fabrication of a Micro-Lens Array Mold by Micro Ball End-Milling and Its Hot Embossing

**DOI:** 10.3390/mi9030096

**Published:** 2018-02-26

**Authors:** Peng Gao, Zhiqiang Liang, Xibin Wang, Tianfeng Zhou, Jiaqing Xie, Shidi Li, Wenhua Shen

**Affiliations:** 1 School of Mechanical Engineering, Beijing Institute of Technology, Beijing 100081, China; 3120140155@bit.edu.cn (P.G.); 3120140161@bit.edu.cn (J.X.); lishidibit@163.com (S.L.); 2 Key Laboratory of Fundamental Science for Advanced Machining, Beijing Institute of Technology, Beijing 100081, China; cutting0@bit.edu.cn (X.W.); zhoutf@bit.edu.cn (T.Z.); 7420161117@bit.edu.cn (W.S.)

**Keywords:** micro-lens array mold, micro ball end-milling, machining strategies, hot embossing

## Abstract

Hot embossing is an efficient technique for manufacturing high-quality micro-lens arrays. The machining quality is significant for hot embossing the micro-lens array mold. This study investigates the effects of micro ball end-milling on the machining quality of AISI H13 tool steel used in the micro-lens array mold. The micro ball end-milling experiments were performed under different machining strategies, and the surface roughness and scallop height of the machined micro-lens array mold are measured. The experimental results showed that a three-dimensional (3D) offset spiral strategy could achieve a higher machining quality in comparison with other strategies assessed in this study. Moreover, the 3D offset spiral strategy is more appropriate for machining the micro-lens array mold. With an increase of the cutting speed and feed rate, the surface roughness of the micro-lens array mold slightly increases, while a small step-over can greatly reduce the surface roughness. In addition, a hot embossing experiment was undertaken, and the obtained results indicated higher-quality production of the micro-lens array mold by the 3D offset spiral strategy.

## 1. Introduction

Micro-lens arrays are widely used throughout the world and they have received increasing growth for a variety of applications [1]. However, fabrication of micro-lens arrays with high accuracy and low cost has been studies by several scholars. The three-dimensional micro glass products mold is difficult, or almost impossible, to be fabricated by simple grinding, for it needs are more sophisticated and axis asymmetric [2]. Hot embossing is a versatile technique to fabricate micro-lens arrays. Additionally, the machining quality of micro-lens array molds is significant for the fabrication process. Micromachining processes are conducted by utilizing micro cutting tools for machining miniature part molds. In addition, micro milling contains several advantages for machining three-dimensional complex microstructures. Moreover, the molding process of micro-lens array should be machined with great surface quality, higher machining accuracy, and uniform consistency. Selecting an appropriate machining strategy would be highly efficient to enhance the machining quality.

The machining strategies and cutting parameters have shown significant impacts on the surface roughness, surface textures, and machining efficiency of micro-milling process. It is noteworthy that the existing machining strategies have different effects on the quality of micro-milling. Hence, adopting a proper machining approach can save time and costs by 88% and 40%, respectively [3]. In traditional milling, several scholars attempted to study the effects of machining methods on the quality of the milling process. Toh [4] proposed a modified offset cutter path strategy for high-speed rough milling of hardened steel. Thepsonthi and Özel [5] presented an approach for optimizing the process parameters in addition to determining the best tool path while conforming to the constraints of micromachining in the micro end-milling of a circular thin-walled rib feature, and the results revealed noticeable improvement in the process by reducing burr formation, as well as enhancing tool life and surface quality. Different machining techniques are proper for various workpiece geometries. Ramos [6] studied different finishing milling strategies (i.e., radiation, parallel lines, and three-dimensional (3D) offset machining) of a complex geometry part containing concave and convex surfaces, and found that the 3D spiral offset strategy is more appropriate for the studied geometry mode. Gologlu et al. [7] investigated the effects of machining strategies on the surface roughness of pocket milling of 1.2738 steel and optimized the corresponding parameters (i.e., cutting speed, feed rate, cutting depth, and step-over). Shaghayegh et al. [8] studied machining strategies for low-curvature convex surfaces and reported that the radial strategy has the best surface texture and the lowest cutting forces compared with 3D offset spiral and radiation approaches. Cardoso et al. [9] optimized the surface roughness by micro-milling and found that a constant overlap spiral strategy has the best surface roughness compared with other techniques used in that study. Krimpen et al. [10] investigated the milling strategies of the sculptured surface and expressed that step-over is the most significant factor on the machining quality of sculptured surface. Souza et al. [11,12] developed a trigonometric equation for the identification of the tool-workpiece contact along the tool path and the point where the tool tip leaves the contact with the workpiece. 

In the micro-molding of micro lens arrays, the effects of different machining strategies on the micro-mold are still unclear. Therefore, the effects of the available machining strategies on the micro ball end-milling of sculpture surfaces should be further investigated. In the fabrication of micro-lens array technology, Xie et al. [13] proposed a novel rapid hot micro-embossing technique utilizing micro-patterned silicon stampers coated with a newly-developed carbide-bonded graphene network to implement rapid heating and cooling. Sun et al. [14] reported a novel droplet-based method for fabricating polymeric micro lenses and lens arrays, and it can lower the manufacturing costs of micro lenses and lens arrays and simplify the manufacturing process.

This study attempts to examine the fabrication of a micro-lens array by micro ball end-milling and the relevant hot embossing processes. The surface morphology is observed, and the surface roughness is measured. In addition, the effects of machining strategies on surface roughness and texture type are investigated. Additionally, the fabrication of a micro-lens array by the hot embossing process is experimentally assessed, and the effects of the machining method on the quality of the micro-lens array produced by hot embossing are evaluated as well.

## 2. Experimental Procedures

**Figure 1:**
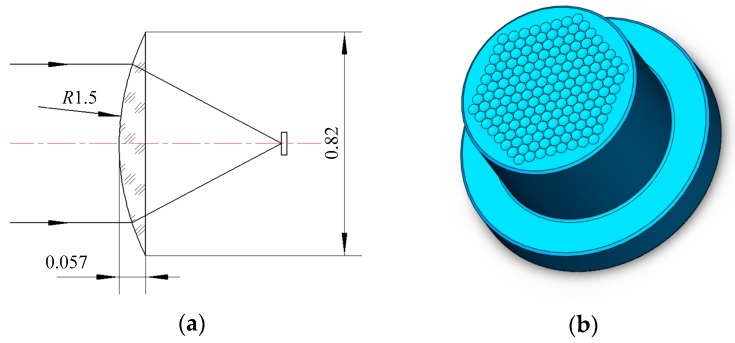
The micro-lens and its array mold. (**a**) Micro-lens unit; and (**b**) micro-lens array mold.

**Figure 2:**
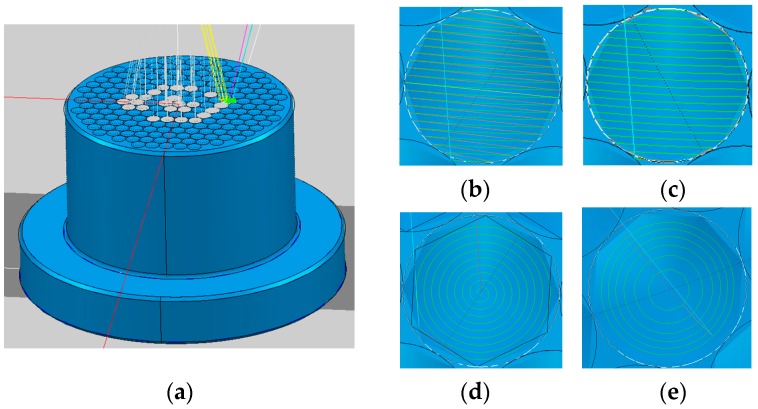
Tool path strategies for machining micro-lens array mold. (**a**) Micro-lens array mold; (**b**) single-direction raster tool paths; (**c**) raster tool paths; (**d**) 3D offset spiral tool paths; and (**e**) equal height finishing tool paths.

The machining process of a concave micro-lens mold is investigated, and the micro-lens mold with a diameter of 0.82 mm is machined by using micro ball end-milling under different machining methods. A micro-lens unit and its array mold are shown in Figure 1. In order to achieve high surface quality, the micro-lens mold is machined under rough and finishing processes. The quality of the finishing machining for the sculpture surface directly affects the quality of hot embossing for fabricating the micro-lens array. In general, the ball end-milling process mainly includes radial, raster, 3D offset, spiral, and equal height finish strategies. Single-direction raster and raster tool paths are parallel linear paths laying on the desired surface as depicted in Figure 2b,c. The step-over distance of the paths is equal in a parametric plane, thus, for steep surface machining, the cutting tool paths are relatively sparse. Compared with the raster method, the single direction strategy can maintain consistent up milling or down milling, however, the raising back-and-forth milling leads to the increase of the time of the machining process. The 3D offset spiral strategy makes a spiral tool path from a given focal point, while maintaining stable contact between cutter and workpiece (Figure 2d). Therefore, the tool paths are composed of offset passes from the geometry in a specified level, horizontally. Several passes are formed, according to the step-over, as well as connecting to each other by a connection link on the surface. The equal height finishing strategy tool paths are conducted based on the sculpture surface machining and maintain a relatively constant height for surface machining, as displayed in Figure 2e.

**Figure 3:**
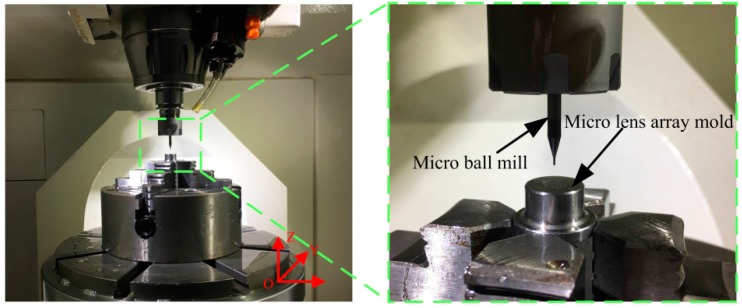
Micro ball end-milling experiment setup.

The experiments are conducted in a five-axis machining center (JDVT600T A13S) (Jingdiao Co., Ltd., Beijing, China), as shown in Figure 3. The micro ball end-mill (KYOCERA Co., Ltd., Kyoto, Japan), with a diameter of 0.5 mm and four cutting edges, is employed. Moreover, AISI H13 hot work steel is utilized, which has shown a proper performance in compression molding. The machining strategies are used for down milling, except raster, which is used for back-and-forth milling. The toolpaths of the milling strategies and numerical control (NC) codes are generated by computer aided manufacturing (CAM) software (Jingdiao Co., Ltd.). Additionally, characteristics of cutting parameters are shown in Table 1.

Initially, a clean-up cut on the workpiece surface is performed with a 10 mm flat end mill to ensure the uniform depth of cut. During rough machining process, a micro ball end-mill with a diameter of 0.5 mm was used under the spiral machining strategy with 0.02 mm machining allowance for AISI H13 tool steel. However, during finishing process, a micro ball end mill is used with a 0.5 mm diameter. During the machining process, cutting oil was used as well. The machined workpiece is cleaned using ultrasonic cleaning machine before measuring. The surface morphology of machined micro-lens mold is measured by a 3D laser scanning microscope (VK-X100, Keyence Co., Ltd., Osaka, Japan). The surface roughness Sa is measured using a non-contact 3D surface profiler (Talysurf CCI Lite, Taylor Hobson Co., Ltd. Leicester, England) and a circle region with a radius of 0.3 mm was sampled at the bottom of the micro-mold.

## 3. Results and Discussion

### 3.1. Surface Morphology of the Micro-Lens Mold

**Figure 4:**
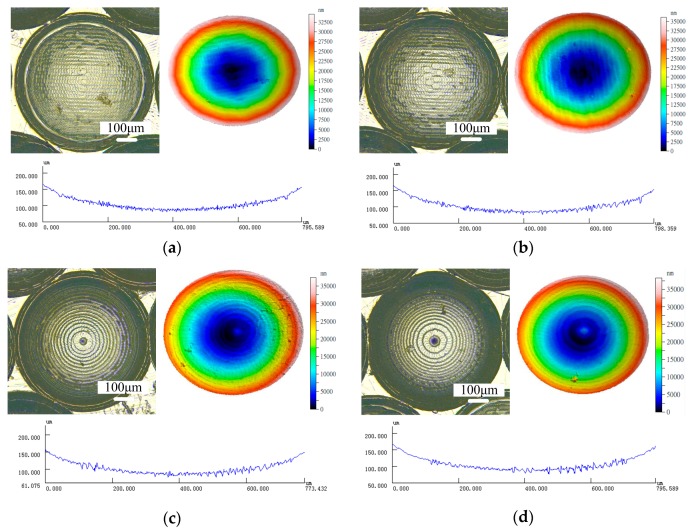
Surface morphology of the machined micro-lens mold under different strategies (*n* = 10,000 r/min, *f* = 45 mm/min, *L_w_* = 0.02 mm). (**a**) Single-direction raster; (**b**) raster; (**c**) 3D offset spiral; and (**d**) equal height finish.

Figure 4 shows the surface morphology of the machined micro-lens array molds in presence of different machining methods. The textures of the micro-lens array molds were machined as well. It can be seen that the micro-lens array molds machined under different strategies represent different markedly texture pattern of crests and gouges on the surface. The crests and gouges on the machined surface are typically made by the step over and feed rate of micro milling. Compared with the single direction raster and raster strategies, there are a number of differences on the textures of the micro-lens array mold machined under 3D offset spiral strategy. The surface morphology of the micro-lens array mold presents a more uniform miscellaneous pattern of crests and gouges. In addition, it can be seen that the surface morphology of the micro-lens array mold machined under the 3D offset spiral strategy contains few burrs at the bottom of the mold compared with that of other two strategies. Developing an optimal machining strategy for micro milling is a significant solution to reduce the burrs. The surfaces of the micro-lens array mold machined under single-direction raster and raster strategies have shown poor surface quality. However, different types of texture patterns for the machined surface were obtained. The surface machined with different types of texture patterns can be used to entrap wear particles and coolant lubrication reservoirs [15]. The micro-lens array mold machined under various strategies demonstrated different values of accuracy at the edge of the mold. It can be seen that the raster strategy is a line at the edge of the mold, and seriously deviates from the design shape. However, the tool paths of the 3D offset spiral strategy are a circle at the edge of the mold. Due to the circle shape of the micro edge, the 3D offset spiral strategy is more appropriate for the micro-mold machining process. 

**Figure 5:**
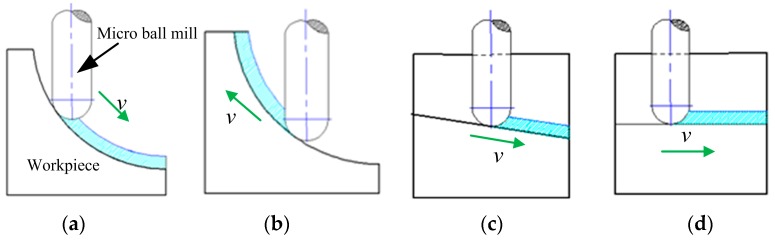
Contact type between the tool and workpiece material. (**a**) Downward cutting; (**b**) upward cutting; (**c**) oblique downward cutting; and (**d**) plane cutting.

The appropriate selection of micro milling strategy is crucial in achieving designed micro-lens array surfaces. Different machining strategies have different machining surface textures due to the cutting direction. The cutting direction is significant effects on the geometric error, and the tool deflection cannot be constant which leads to the uniform geometric errors of machined surface [12]. Therefore, the direction of cutting should be taken account to investigate the cutting performance of different machining strategies. According to the shape of the micro-lens mold, the machining direction was mainly divided into downward cutting, upward cutting, oblique downward cutting, and plane cutting as shown in Figure 5. The single direction raster and raster strategies are downward cutting and upward cutting from the sculpture surface. The 3D offset spiral strategy cutting direction is oblique downward cutting and the equal height finish strategy cutting direction is parallel to the tool path. It can be seen that the sculpture surface machined with different strategies generates different scallop heights and the offset spiral strategy has a smaller scallop height in the machining surface.

**Figure 6:**
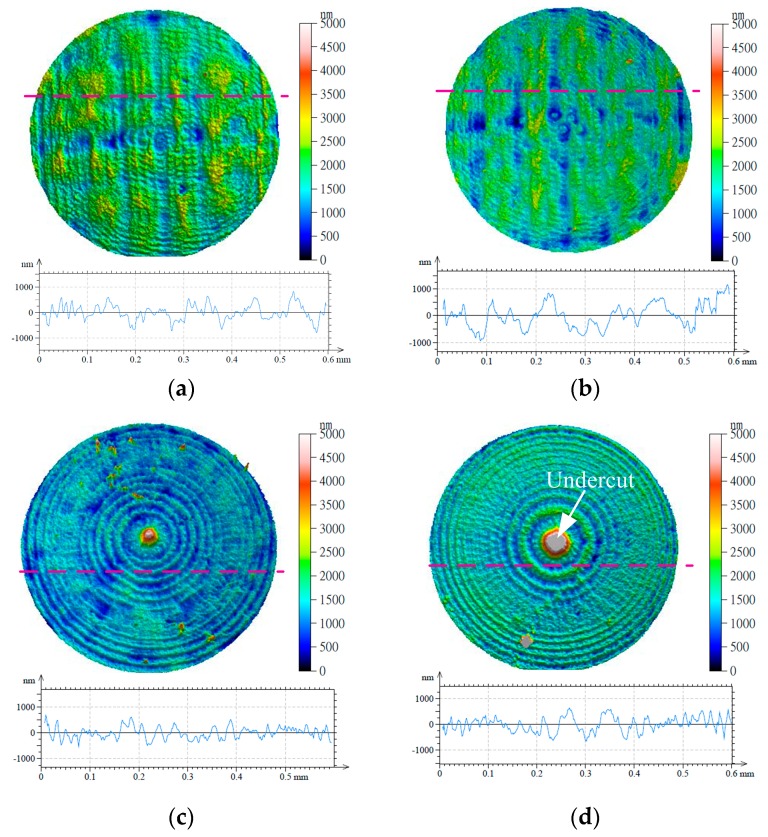
Surface morphology and outline of micro-molds with different machining strategies (*n* = 10,000 r/min, *f* = 45 mm/min, *L_w_* = 0.02 mm). (**a**) Single-direction raster; (**b**) raster; (**c**) 3D offset spiral; and (**d**) equal height finish.

Figure 6 shows the surface morphology and outline of the micro-lens array mold with different machining strategies. It can be seen that the single direction raster and raster machining strategies represent the maximum scallop height, while the 3D offset spiral strategy contains the minimum scallop height. However, the 3D offset spiral strategy and the equal height finish strategy appear undercut at the bottom of the micro-mold. It can also be seen that the maximum value scallop heights of the micro-mold machining with the single-direction raster, raster, 3D offset spiral and equal height finish strategies are about 0.9 μm, 1 μm, 0.6 μm, and 0.8 μm, respectively.

### 3.2. Surface Roughness

#### 3.2.1. Effects of Tool Path Strategies on Surface Roughness

**Figure 7:**
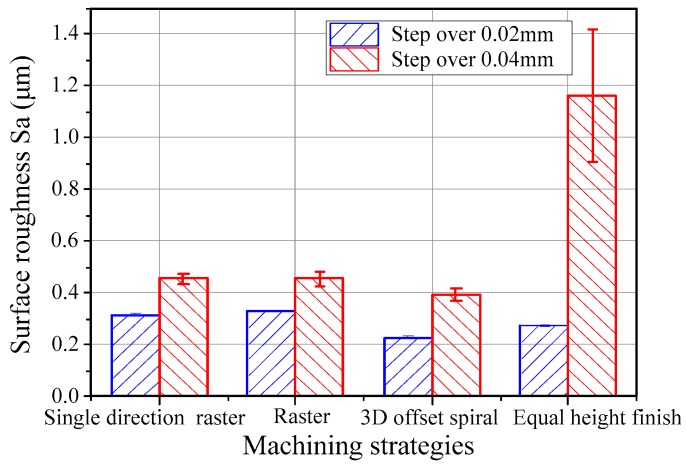
Effects of machining strategies on the surface roughness of a micro-lens array mold.

Figure 7 illustrates the surface roughness of the micro-lens array mold machined under the single-direction raster, raster, 3D offset spiral, and equal height finish strategies. It can be seen that the micro-lens array mold machined under the single direction raster and raster strategies have higher surface roughness with a step-over of 0.02 mm in comparison with that of other approaches. The surface roughness of the micro-lens array mold machined under the 3D offset spiral strategy is minimal, while the micro-lens array mold machined with the equal height finish strategy has the highest surface roughness with a step-over of 0.04 mm. This can be justified in that the tool paths of the single-direction raster and raster strategies are discontinuous, and ascendant and descendant cutting appear during the machining of the sculpture surface, leading to the generation of machining vibrations.

**Figure 8:**
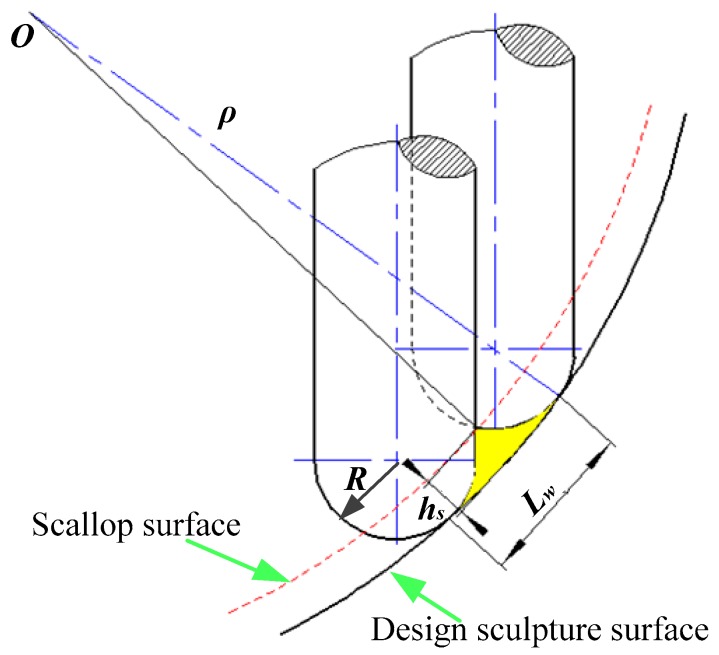
Calculation scheme of the scallop height.

The mathematical formulation of the toolpath step-over can be derived as a function of the radius of the cutter, the scallop height, and the radius of the surface curvature. The radius of the tool path, step-over, and radius of surface curvature are given as constants. It should be mentioned that the surface theory scallop height does not change during machining process. The calculation of the theoretical scallop height is shown in the Figure 8. The scallop height of the sculpture surface forms the scallop surface and deviates from the designed sculpture surface. The scallop height, *h_s_*, as a function of concave surfaces, can be given by [16]:

(1) $$h_{s}=\rho -(\rho -R)\sqrt{1-{\left(\frac{L_{w}}{2\rho }\right)}^{2}}-\sqrt{R^{2}-{\left[\frac{(\rho -R)L_{w}}{2\rho }\right]}^{2}}$$

where *R* is the radius of the micro ball mill, *L_w_* denotes the step-over, and *ρ* is the radius of the sculpture surface.

According the calculation procedure of scallop height, when the step over of tool path *L_w_* is 0.02 mm, the theoretical value of scallop height is 0.1667 μm. However, the experimental scallop height considerably deviates from theoretical scallop height compared with traditional milling methods. In traditional milling, the experimental scallop height of complex freedom surface is lower or closer to the theoretical one [17]. The reason is that during the micro milling, the micro tool easily produces size effect, while ploughing behavior occurs, instead of shearing. Additionally, compared with traditional milling, the diameter of the micro mill is smaller and the tool deflection occurs during the milling. Due to the tool deflection and tool run out, the position of the micro mill is easy deviated, and the tool deformation can be expressed in [18]; the tool paths of 3D offset radial strategy can control the scallop height in complex freeform surface milling [16]. Moreover, micro milling is more sensitive to environmental vibrations, leading to poor surface quality of the micro-lens array mold. During the micro ball end-milling, the sculpture surface of the micro-lens array mold easily produces burrs at the bottom of the surface, and the experimental scallop height deviates from theoretical scallop height. It can be concluded that the 3D offset spiral technique is more appropriate for machining some parts with complex surfaces.

#### 3.2.2. Effects of Cutting Speed and Feed Rate on Surface Roughness

**Figure 9:**
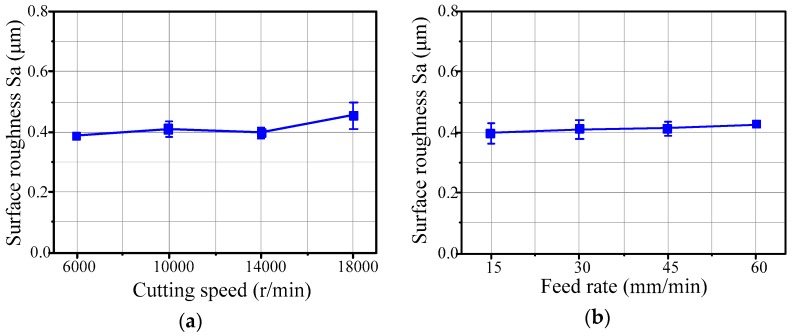
(**a**) Effects of spindle speed on surface roughness (*f* = 45 mm/min); and (**b**) the effects of the feed rate on surface roughness (*n* = 10,000 r/min).

Figure 9 illustrates the effects of cutting speed and feed rate on the surface roughness of the micro-lens array mold machined by the 3D offset spiral strategy. The step-over of the tool path is 0.04 mm, obtained by experimental testing. It can be seen that the cutting speed and the feed rate have slight effects on the surface roughness of the micro-lens array mold. With an increase in the cutting speed, the surface roughness of the micro-lens array mold slightly increases as well. However, the surface roughness of the machined micro-lens array mold obviously increases with the cutting speed ranging from 14,000 to 18,000 r/min, as shown in Figure 9a. Furthermore, the surface roughness of the micro-lens array mold slightly increases with increase of the feed rate (see Figure 9b). The reason is that the machining vibrations will increase in the presence of the higher cutting speed. Compared with macro milling, the micro milling is more sensitive to machining vibrations and tool deflection. The machining vibrations of micro milling amplify the tool deflection and tool run out with the increase of the cutting speed. This produces a higher scallop height and deteriorates the machining quality of the micro-lens array mold. Due to the size effect, the surface roughness of the machined micro-lens array mold does not clearly increase with the increase of feed rate.

### 3.3. Hot Embossing of the Micro-Lens Array

**Figure 10:**
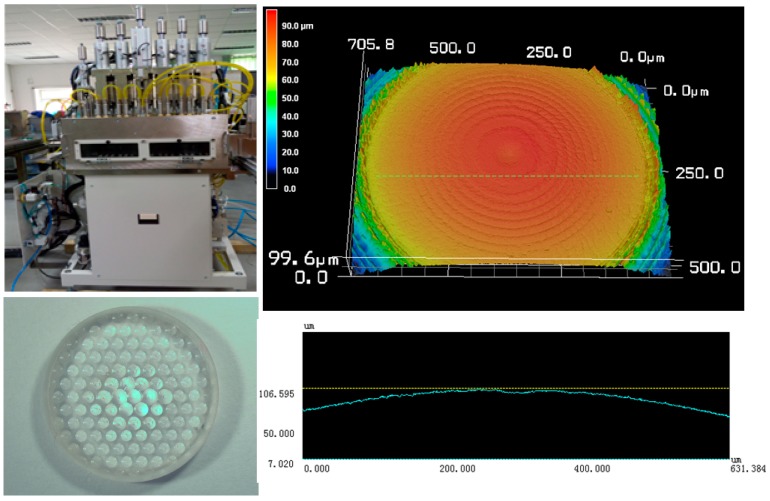
Experimental setup for hot embossing the micro-lens array and the achieved results.

Hot embossing is an efficient technique to fabricate glass microstructures. Wang [19] fabricated glass microstructures using hot embossing, and the glass molding process of cuboid micro protrusions was investigated. The experimental procedure for hot embossing of micro-lens array was conducted using an ultra-precision injection molding machine (PFLF7-60A) (SYS Co., Ltd., Osaka, Japan). A particular type of glass K-PG375 (SUMITA Optical Glass, Inc., Saitama, Japan) is used for the hot embossing process, and the optical glass mechanical and thermal properties is shown in Table 2. During the experiment, nitrogen gas was used to protect the micro-lens molds from oxidation. The hot embossing parameters are temperature 390 °C and compression pressure 0.1 MPa. The single pressing time is 60 seconds in fabricating the micro-lens array mold. After performing the hot embossing, the fabricated micro-lens array with microstructure glass was measured by a 3D laser scanning microscope. The description of experimental setup for hot embossing the micro-lens array and the achieved results are displayed in Figure 10.

**Figure 11:**
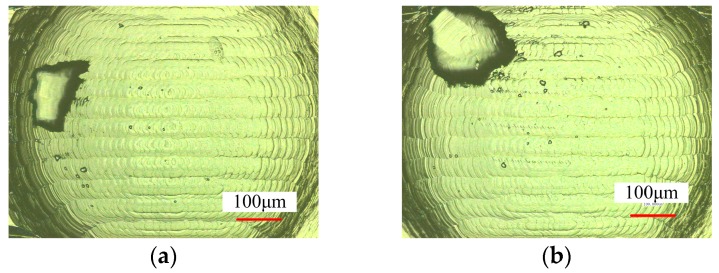

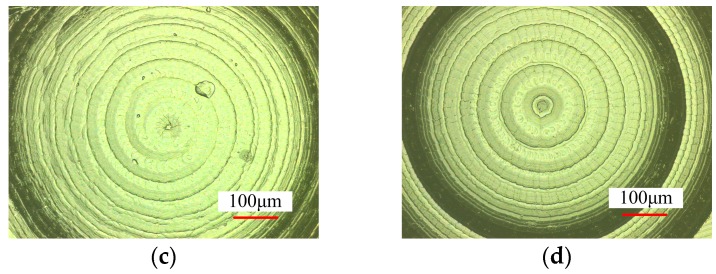
Surface morphology of the hot embossed micro-lens array mold. (**a**) Single direction raster; (**b**) raster; (**c**) 3D offset spiral; and (**d**) equal height finish.

The hot embossing of micro-lens array conducted experimentally indicated that the micro ball end-milling is an effective technique to machine the micro-lens array. Figure 11 shows the results of surface morphology of the hot embossed micro-lens with the micro-mold machining at *n* = 10,000 r/min, *f* = 45 mm/min, *L_w_* = 0.04 mm. It can be seen that the micro-textured molds is very clear, and the surface of micro-lens array mold machined by the 3D offset spiral strategy is smoother than that of other strategies. The different types of texture patterns for the micro-lens array mold can be fabricated by a method with different functions. The experimental results indicated the micro-lens array mold machined under the 3D offset spiral strategy shows a higher-quality production of the micro-lens array.

**Figure 12:**
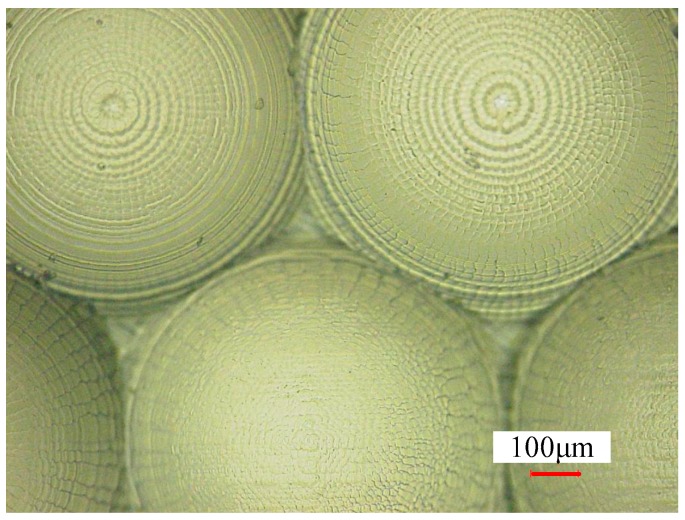
Surface morphology of the micro-lens array under different hot embossing parameters.

Figure 12 shows the surface morphology of the micro-lens array hot embossed with the micro-lens mold machining at *n* = 10,000 r/min, *f* = 45 mm/min, *L_w_* = 0.02 mm. It can be seen that the hot embossing’s parameters have significant impacts on the quality of micro-lens array. The hot embossing of the micro-lens array under a temperature and pressure of 390 °C and 0.1 MPa is smooth and clear. Surface texturing demonstrated appropriate performance from boundary lubrication to hydrodynamic lubrication [15]. Surface texturing is used in several applications, such as magnetic surface storage devices, sliding contact elements, rolls of forming processes, hydrodynamic bearings, and mechanical face seals to improve the friction properties [15,20]. In addition, various machining strategies can prepare different types of textures, and then the surface structures can be hot embossed by the glass products. Moreover, different surface texture type microstructures can be generated well by controlling the machining strategies, feed rate, and step-over.

## 4. Conclusions

The effects of machining strategies on the quality of a micro-lens array mold are investigated. The tool paths of the machining strategies are generated by CAM. Additionally, machining a micro-lens array mold is experimentally performed in which the surface morphology, surface roughness, as well as scallop height were measured. According to the achieved results, the following conclusions are drawn:

(1) Compared with traditional milling, the micro ball end-milling strategy has a greater effect on the workpiece surface quality in machining. The sculpture surface machining of the micro-lens array mold produced burrs more easily, and the experimental scallop height seriously deviated from the theoretical scallop height.
(2) The 3D offset spiral approach has homogeneous tool paths, and the experimental results showed that the 3D offset spiral strategy has a better quality of machining. Additionally, high-precision machining at the edge of the micro-lens array mold was obtained using a 3D offset spiral strategy, with minimum surface roughness. It is worth mentioning that the 3D offset spiral strategy is more appropriate for the machining process of concave micro-lens array molds. With an increase of the cutting speed and feed rate, the surface roughness of the micro-lens array mold increases slightly, while a small step-over can greatly reduce the surface roughness.
(3) Eventually, the micro-lens array was experimentally investigated by hot embossing and the results indicated that a higher quality production of the micro-lens array could be obtained by the 3D offset spiral strategy.

## Figures and Tables

**Table 1:** Experimental cutting parameters.

Parameter	Spindle Speed *n* (r/min)	Feed Rate *f* (mm/min)	Step over *L_w_* (mm)	Depth of Cut *a_p_* (μm)	Coolant
Level	6000/10,000/14,000/18,000	15/30/45/60	0.02/0.04	20	Oil metal fluid

**Table 2:** Optical glass mechanical and thermal properties.

**Mechanical Properties**	**Vickers Hardness Hv**	**Young’s Modulus (N·m^−2^)**	**Poisson Rate *σ***	**Modulus of Rigidity (N·m^−2^)**
Level	428	625 × 10^8^	0.252	249 × 10^8^
**Thermal Properties**	**Transformation Point (°C)**	**Yielding Point (°C)**	**Thermal Expansion (°C^−1^)**	**Thermal Conductivity (W·m^−1^·K^−1^)**
Level	344	367	129 × 10^−7^ (−30~+70 °C)	0.688
